# What Is the Relationship between the Chewing Ability and Nutritional Status of the Elderly in Korea?

**DOI:** 10.3390/nu15092042

**Published:** 2023-04-23

**Authors:** Sohye Kim, Yong-Seok Kwon, Kyung Hee Hong

**Affiliations:** 1 Nutrition Care Services, Seoul National University of Bundang Hospital, Seongnam 13620, Republic of Korea; 2 Department of Medical Nutrition, Graduate School of East-West Science, Kyung Hee University, Yongin 17104, Republic of Korea; 3 Department of Agro-Food Resources, National Institute of Agricultural Sciences, Wanju 55365, Republic of Korea; 4 Department of Food Science and Nutrition, Dongseo University, Busan 47011, Republic of Korea

**Keywords:** chewing ability, dietary intake, nutritional status, elderly, KNHANES

## Abstract

This study aims to determine the relationship between chewing ability and the nutritional status of the elderly in Korea. This study utilized the data from the Korea National Health and Nutrition Examination Survey (KNHANES) conducted from 2013–2018 for persons who were ≥65 years of age. Of the 7835 subjects, 43.2% had chewing difficulty. Compared to the normal group, the chewing difficulty group had more stress, lower exercise frequency, less snack intake, a lower frequency of eating out, and a higher proportion of food insecurity. The chewing difficulty group had significantly lower food intake compared to the normal group, including various food groups such as cereals and grain, potatoes, fruits, meat, and milks and dairy products. The intake of fresh fruits was 24.5% lower and the intake of plant food (fresh fruits and nonstarchy vegetables) was 17.8% lower in the chewing difficulty group compared to the normal group. In addition, the intake of most nutrients (carbohydrates, fat, calcium, phosphorus, sodium, potassium, vitamin A, riboflavin, niacin, and vitamin C) was significantly lower in the chewing difficulty group than in the normal group. The chewing difficulty was significantly associated with undernutrition (OR = 1.63). In conclusion, chewing ability is closely related to food and nutrient intake among the elderly, which can decrease the quantity and quality of diet and is also related to undernutrition. Therefore, it is necessary to develop customized nutrition programs and aging-friendly food products that consider the chewing ability of the elderly.

## 1. Introduction

The improvement in living conditions and the development of medical technology have led to a rapid increase in the elderly population worldwide. The UN’s World Population Prospects 2022 predicts the proportion of the global population aged ≥65 years is expected to increase from 10% in 2022 to 16% by 2050 [1]. In Korea, the elderly population comprised 17.5% of the entire population in 2022 and it is predicted that by 2025, Korea will become a superaged society as one in five people will be elderly citizens; the elderly population is predicted to increase up to 40.1% by 2050 [2]. As the elderly population increases, problems related to elderly people have emerged as important social issues. In particular, interest in the health and quality of life caused by physical aging has received increased attention.

Due to the physical and mental changes that occur with aging, the elderly often experience difficulties in their daily lives. Although maintaining a balanced nutritional status through adequate dietary intake is vital to achieving a healthy lifestyle among the elderly, their chewing ability will decline due to problems such as tooth loss, which directly affects food intake [3,4]. In fact, about 40% of the elderly population in Korea reportedly experiences chewing difficulty [5].

As the first step in digestion, chewing is the action of crushing and mixing consumed food, and chewing difficulty causes indigestion and malabsorption [6]. In addition, chewing difficulty creates a tendency to have a diet centered on easy-to-chew food, which leads to declines in the quantity and quality of the diet, which may lead to nutritional imbalances [3]. Elderly people with chewing difficulties eat less difficult-to-chew foods such as meat, vegetables, and fruits [7,8,9], and the quality of their diet was lowered due to a lack of variety in the types of foods [10,11]. Furthermore, elderly people with chewing difficulties have a low nutritional status score [12] and a high nutritional insufficiency risk level [7,13]. These previous studies support the idea that the chewing ability of the elderly is an important factor in maintaining a proper diet and good nutrition.

Dietary and nutritional problems caused by chewing difficulty in the elderly can cause declines in quality of life for the elderly and deteriorate their health by acting as obstacles to the maintenance of physical and mental health. Decreased chewing ability lowers the related quality of life [5,14], affects mental health issues such as increasing the incidence of depression [5,14,15], and has a demonstrated association with chronic diseases such as musculoskeletal disease and respiratory disease [16]. Chewing ability is an important factor influencing the quality of life and health in relation to nutritional status. Reportedly, improving the chewing ability in the elderly affects nutritional status and improves both cognitive functions [12] and quality of life [17,18,19]. Conversely, the elderly with reduced chewing ability showed an increased mortality risk accompanied by reduced dietary diversity [11].

In a study of the French elderly, the odds of malnutrition decreased by 15% when chewing difficulty decreased by 10 points [20]. In a study of the Japanese elderly, chewing difficulty increased the risk of undernutrition by 1.5 times [9]. There have been studies on food and nutrient intake status depending on the chewing ability of the Korean elderly using data from the Korean National Health and Nutrition Examination Survey (KNHANES) [7,8,21]. However, those reports were based on the results of analyzing pre-2015 data, and there have been few studies on the relationship between chewing ability and nutritional status.

As aging is rapidly progressing in Korea, this study aims to investigate the relationship between the chewing ability and nutritional status of the Korean elderly based on comprehensive data in the KNHANES 6th (2013–2015) and 7th (2016–2018).

## 2. Materials and Methods

### 2.1. Study Population and Design

This study utilized raw data from the Korean National Health and Nutrition Examination Survey (KNHANES) conducted in 2013–2018. The primary survey subjects were elderly people ≥65 years old who participated in the health survey, oral examination survey, and dietary survey (24-h recall survey) (n = 9465). Of these, subjects with a total caloric intake of <500 kcal or >5000 kcal per day (n = 146) and subjects with missing data from the dietary survey (n = 1484) were excluded from the analysis. As a result, the final total selected study subjects were 7835 persons. The subjects were classified into two groups based on their subjective chewing discomfort level: subjects who responded “very uncomfortable” or “uncomfortable” were in the “chewing difficulty” group and those who responded “moderate”, “not uncomfortable”, or “not uncomfortable at all” were grouped in the “normal” group. The two groups were compared and analyzed in terms of their chewing ability.

### 2.2. General Characteristics

The analysis was conducted with reference to the survey subjects’ gender, age, marital status, education level, residential region, employment status, and income level. Age was classified into two groups (65–74 years old and >75 years old), marital status was classified as single or married, and education level was classified into three categories (less than a high-school diploma, a high-school diploma, or college degree or higher). The residential region field was classified into city or rural area using dong/eup/myeon, and employment status was divided into employed or unemployed. Household income level was divided into upper, middle-upper, middle-lower, and lower.

### 2.3. Health Behavior

Questions related to health behavior were smoking status, alcohol consumption level, stress level, exercise frequency, and weight status. Smoking status was divided into “non-smoker”, “past smoker”, and “current smoker”; alcohol consumption level was classified into four categories based on the frequency of drinking (4 or more times/week, 2–3 times/week, 1–4 times/month, or less than 1 time/month). The stress level was divided into four categories (“severe stress”, “moderate stress”, “mild stress”, or “no stress”). Four categories were assigned for exercise frequency (less than 1 day/week, 1–2 days/week, 3–4 days/week, or more than 5 days/week). Weight status was classified using the BMI (kg/m^2^) index; a BMI of <18.5 kg/m^2^ was classified as underweight, 18.5–23.0 kg/m^2^ as normal, 23.0–25.0 kg/m^2^ as overweight, and ≥25.0 kg/m^2^ as obese.

### 2.4. Dietary Behavior

Five questions investigated dietary behaviors: breakfast frequency, snack intake, serving location of the meal, frequency of eating out, and food security. Breakfast frequency was classified as 5–7 times/week, 3–4 times/week, 1–2 times/week, or rarely. The snack intake answers were either yes or no. For the serving location of meals, home, commercial locations, and institution locations were each classified as yes or no. Eating-out frequency was divided into six categories: more than 1 time/day, 5–6 times/ week, 3–4 times/week, 1–2 times/week, 1–3 times/month, and rarely. Food security was classified as enough food secure, mild, moderate, or severe food insecure depending on the response to the KNHANES question “which of the following best describes your household’s eating habits over the past year?”.

### 2.5. Food and Nutrient Intake

The dietary intake survey in the KNHANES was conducted using a 24-h recall method in which all food intake contents were for one day before the survey was answered. Food intake was categorized into 17 food groups (cereals and grain products, potatoes and starches, sugars and sweets, legumes and their products, seeds and nuts, vegetables, mushrooms, fruits, meat, poultry and their products, eggs, fish and shellfish, seaweeds, milks and dairy products, oils and fats, beverages, seasonings, and other food). The intake of each food group was calculated, and the subjects’ total food intake was calculated. In addition, the total intake of fresh fruits (excluding jams, sugar-preserved fruits, and fruit juice) and the total nonstarchy vegetable intake (excluding salted vegetables and vegetable juice) were calculated [22]. For comparison with the intake standard recommended by the World Cancer Research Fund (WCRF), the combined intake of fresh fruits and nonstarchy vegetables was obtained [23]. In addition, energy, macronutrients (carbohydrate, protein, and fat), and micronutrients (calcium, phosphorus, iron, sodium, potassium, vitamin A, thiamine, riboflavin, niacin, and vitamin C) intake were analyzed and the energy distribution ratio of macronutrients (carbohydrate, protein, and fat) was calculated. According to the nutritional insufficiency criteria of KNHANES, the subjects with an energy intake <75% of their estimated energy requirement (EER), and intakes of calcium, iron, vitamin A, and riboflavin less than the estimated average requirement (EAR) were classified as undernutrition.

### 2.6. Statistical Analysis

The KNHANES data was obtained by stratified multistage sampling rather than a simple random sampling method. The collected data was analyzed with a consideration of weight, strata variable (KSTRATA), and cluster variable (Primary Sampling Unit, PSU). All analyses were performed using the Statistical Analysis System (SAS) (ver. 9.4, SAS Institute, Cary, NC, USA). Frequency analysis was performed on general characteristics, health-related questionnaires, and dietary-related questionnaires. The results were shown as percentages (weighted percentage) considering frequency and weight. The results of analyses on the intake of food and nutrients, and plant food were presented as means and standard errors using the Surveymean procedure. The Surveyreg procedure was used for the significance test: the *t*-test was used when no correction was made, and a general linear model was used when the correction was performed. Gender, age, and energy intake were used as correction variables. The correlation between chewing ability and nutritional insufficiency was presented as an odds ratio (ORs) and 95% confidence interval (CI) by performing logistic regression analysis through the Surveylogistic procedure. In addition, the influences based on gender, age, smoking status, drinking, stress level, weight status, intake of milks and dairy products, food security, snacking, eating out and breakfast frequencies, education level, household income, and marital status were adjusted in stages prior to multiple logistic regression analysis.

## 3. Results

### 3.1. General Characteristics

Table 1 shows the survey subjects’ general characteristics. Of the 7835 subjects, 56.8% (n = 4378) answered that they had no chewing difficulty and 43.2% (n = 3457) answered that they had chewing difficulty. In the chewing difficulty group, the proportion of the following three categories was higher: women (*p* < 0.001), elderly >75 years old (*p* < 0.001), and residents living in rural areas (*p* = 0.005). The education and income levels of the chewing difficulty group were lower than those of the normal group (*p* < 0.001 and *p* < 0.001, respectively). There was no significant difference in either marital or employment statuses between the two groups.

### 3.2. Health-Related Factors

Table 2 presents the results of the health-related factors. The proportion of smokers in the chewing difficulty group was 12.3%, which was higher than that of the normal group (8.9%) (*p* < 0.001), and the frequency of drinking was lower in the chewing difficulty group (*p* = 0.002). The chewing difficulty group expressed higher levels of stress: 25.5% of the chewing difficulty group and 13.7% of the normal group responded as “severe stress” or “moderate stress” (*p* < 0.001). Exercise frequency was lower in the chewing-difficulty group: 11.3% of the chewing-difficulty group and 18.6% of the normal group reported exercising more than 3–4 times/week (*p* < 0.001). The proportion of underweight was higher and the overweight was lower (*p* = 0.001) in the chewing difficulty group.

### 3.3. Dietary Behaviors

Table 3 shows the results of the analysis of dietary behaviors according to chewing ability. There was no significant difference in the breakfast frequency and meals at the institution location between the two groups, however, the proportion of snacking, meals at a commercial location, and eating out were lower in the chewing difficulty group than in the normal group (*p* < 0.001). In particular, the proportion of eating out rarely was 28.2% in the chewing difficulty group, which was higher than 18.7% in the normal group. The proportion of food insecurity was higher in the chewing difficulty group; the proportion of those who suffered either moderate or severe food insecurity in the chewing difficulty group was 8.6%, twice the rate of the normal group (4.3%) (*p* < 0.001).

### 3.4. Food Group Intakes

Table 4 shows the results of the food intake analysis between the two groups. The total food intake was significantly lower in the chewing difficulty group compared to the normal group (unadjusted *p*-value = 0.044; adjusted *p*-value = 0.017). The intake of various food groups was significantly lower. The results after adjusting for gender, age, energy-intake factors, intake of “cereals and grain products”, “potatoes and starches”, “fruits,” “meat, poultry and their products” and “milks and dairy products” were lower in the chewing difficulty group (*p* = 0.022, *p* = 0.012, *p* < 0.001, *p* = 0.042, and *p* = 0.029, respectively). In addition, in the uncorrected analysis results, the intake of seaweeds, eggs, and beverages was lower in the chewing difficulty group than in the normal group (*p* = 0.037, *p* = 0.020, and *p* = 0.049, respectively).

### 3.5. Plant Food (Fresh Fruits and Nonstarchy Vegetables) Intake

Table 5 shows the results of analyzing the intake of plant food, i.e., fresh fruits and nonstarchy vegetables. In KNHANES, the fruits category included fruit jam and fruit juice, and the vegetables category included salted vegetables and vegetable juice along with kimchi. In the present study, data excluding jam, fruit juice, and salted vegetables were used in the analysis. The intake of nonstarchy vegetables was not significant, though the fresh fruits intake was 24.5% lower (adjusted *p*-value = 0.015) and the intake of fresh fruits and nonstarchy vegetables was also 17.8% lower (adjusted *p*-value < 0.001) in the chewing difficulty group, compared to the normal group.

### 3.6. Nutrient Intakes

Table 6 presents the results of nutrient intake according to chewing ability. There was no statistically significant difference in energy intake between the two groups, though the chewing difficulty group tended to intake less energy than the normal group. In the chewing difficulty group, the carbohydrate intake was 4.8% lower (unadjusted *p*-value = 0.042) and fat intake was 9.2% lower (adjusted *p*-value = 0.035) compared to the normal group. The results of the analysis with adjustment for gender, age, and energy intake showed that the intake of minerals except for iron (calcium, phosphorus, sodium, and potassium), and vitamins except for thiamine (vitamin A, riboflavin, niacin, and vitamin C) were lower in the chewing difficulty group than in the normal group. The energy distribution of carbohydrates, proteins, and fat did not show a significant difference between the two groups.

### 3.7. Relationship between Chewing Ability and Undernutrition

Table 7 presents the association between chewing ability and undernutrition. Chewing difficulty has a shown association with undernutrition (OR = 1.630, 95% CI = 1.354–1.961, *p* < 0.001, model 1); the proportion of undernutrition (energy intake < 75% of EER and the intake of calcium, iron, vitamin A, and riboflavin less than EAR) in the chewing difficulty group was 11.6% (n = 416), which was 1.5 times higher than that of the normal group at 7.53% (n = 322) (*p* < 0.001). The chewing difficulty was found to be independently and significantly correlated with, even when controlling for gender, age, smoking, drinking, stress, weight status, milks and dairy products intake, food security, snack, eating-out frequency, breakfast, education, income, and marital status (OR = 1.332, 95% CI = 1.082–1.615, *p* < 0.01, model 4). Moreover, chewing ability was significantly correlated with an insufficiency of energy and nutrients such as calcium, iron, vitamin A, and riboflavin.

## 4. Discussion

As the elderly population is rapidly increasing, maintaining a healthy life for the elderly has become an important social issue. Unfortunately, for many elderly people, it is difficult to improve their quality of life and maintain health in old age through adequate diet due to chewing problems. In this study, the relationship between dietary behavior, food intake characteristics, and nutritional status according to the chewing ability of the elderly was investigated.

The result of KNHANES 2013–2018 analysis showed that the proportion of elderly people who had chewing difficulty was 43.2%, which showed a decreasing trend compared to the results for 2013 (47.7%) [8] and 2007–2010 (54.3%) [7]. However, it is obvious that a significant number of elderly people suffer from chewing difficulty, suggesting that chewing ability is an important issue in the health of the elderly. This study shows that the proportion of the elderly with chewing difficulty was higher in women than in men, which is thought to be due to bone loss and connective tissue breakdown caused by estrogen deficiency after menopause, which can lead to tooth loss in women [24]. In addition, this study confirmed the results of previous studies [5,7,8] that the chewing difficulty rate increases as aging since the dental condition gradually deteriorates with age. As in previous studies [25,26], socioeconomic factors such as education and income are closely related to oral health; in this study, the education and income levels of the elderly with chewing difficulty were lower.

This study showed that the chewing difficulty group had a higher smoking rate and lower alcohol consumption frequency. This result is not congruent with Park et al. [7], which showed no association between chewing ability and smoking or drinking based on data analysis of KNHANES 2007–2010. In this study, the elderly with chewing difficulty had a higher level of stress perception and a lower frequency of exercise. This result is consistent with the results of a study on adults who found that when chewing was uncomfortable, they felt more stressed and had lower exercise ability and physical activity [14]. Along with these aspects, chewing difficulty also reportedly affects mental health, such as increasing depression, and lowering the health-related quality of life [5,14,15,27]. In addition, the prevalence of metabolic chronic diseases such as diabetes and cardiovascular disease was found to be associated in the elderly with chewing difficulty [16]. Smoking, lack of exercise, and increased stress in the elderly with chewing difficulty are thought to be risk factors that aggravate aging-related health problems and increase the risk of chronic diseases.

There was no difference in the frequency of breakfast between the two groups, but the snack intake and frequency of eating out were lower in the chewing difficulty group. It is thought that chewing difficulty can reduce the variety of food intake in the elderly, thereby reducing the quality of their meals. In previous studies, the elderly with chewing difficulty consumed fewer side dishes [7] and had a low dietary diversity score [10,11], whereas the nutritional status of those who snacking was relatively good [28]. These results support that chewing difficulty is a risk factor for a poor diet. Moreover, in this study, the chewing difficulty group had a higher proportion of food insecurity, which is related to the result that shows a higher occurrence of chewing difficulty among the elderly with low income. It is expected that food insecurity, combined with low-income status, can negatively affect the diet quality of the elderly with chewing difficulty.

The results of food intake analysis according to chewing ability in the present study are similar to previous studies that analyzed KNHANES [7,8] and a study in Japan [9]: the elderly with chewing difficulty had less total food intake and intake of most food groups such as cereals and grains, potatoes, meat, poultry, and eggs. In this study, the chewing difficulty group also consumed less milk and beverages, which does not need to be chewed. However, a study of the Greek elderly found that the consumption of easy-to-chew soft foods (chicken, fish, grains, and dairy products) was higher among the elderly with chewing difficulty [29], and Kwon et al. [8] reported that the elderly Koreans with chewing difficulty consumed a greater amount of beverages. Therefore, we suggest that efforts should be made to encourage the consumption of beverages including milk and yogurt, which can help improve the bone health of the elderly.

In previous studies, the intake of difficult-to-chew food such as vegetables and fruits was significantly lower in the elderly, especially those with chewing difficulty [7,8,9]. However, in this study, we found that fruit intake was low in the chewing difficulty group, though there was no significant difference in vegetable intake. The low intake of fruits in the chewing-difficulty group is thought to be related to their low intake of snacks. In the food group classification of KNHANES, vegetables include various types of kimchi, which are basically consumed in the diets of the Korean elderly, to be considered when interpreting the results.

The intake of fresh fruits, excluding fruits preserved in sugar, jam, and fruit juice was 24.5% lower in the chewing difficulty group than in the normal group, and the combined intake of fresh fruits and nonstarchy vegetables was also significantly lower in the chewing difficulty group. Elderly people with many missing teeth had a low preference for vegetables and fruits [30]. Poor chewing ability is thought to limit the intake of hard or tough plant food, which is a factor leading to insufficient intake of various micronutrients and dietary fiber. In particular, the results of this study indicated that the daily intake of fresh fruits and nonstarchy vegetables for the elderly with chewing difficulties was 334.82 g, which was only 83.7% of the minimum daily intake of 400 g recommended by the WCRF [23]. Reportedly, dietary diversity and fruit intake may also play a role in mediating a possible mortality effect of chewing ability, in particular, the mortality rate of the elderly who have metabolic syndrome with chewing discomfort is more than doubled [11]. These results suggest that more various plant food should be consumed for the prevention and management of metabolic disease in the elderly. Since elderly people with reduced chewing ability naturally avoid foods that are difficult to chew, the range of food choices is naturally limited [20]. In a study in Taiwan [11], similar to this study, the fruit intake of the elderly with chewing difficulty was low, whereas Israeli elderly people with chewing difficulty consumed fewer vegetables but showed no significant difference in fruit intake [31]. The discrepancy in the results can be interpreted as showing that the dietary patterns of the elderly differ depending on dietary cultures, and the effect of chewing difficulty on changes in food consumption may differ. The result that there was no difference in vegetable intake related to chewing ability in this study may reflect the efforts of some elderly people with chewing difficulty to eat more vegetables. It can be inferred that traditional Korean cooking methods that incorporate moisture and heat such as soup, steaming, and boiling, which are familiar to the Korean elderly, soften vegetables and make them comfortable to eat. In addition, among fruits, the elderly who had difficulty chewing consumed soft and ripe persimmon [7]. These results suggest that it is necessary to encourage the consumption of plant foods more actively by finding ways of making it easier for the elderly with chewing difficulty to consume plant foods. In fact, in Greece, various food preparation methods reportedly helped the elderly with chewing difficulty overcome eating problems, and chewing difficulty did not affect the frequency of consuming difficult-to-chew foods such as meat, fruits, and vegetables [29]. The results of this study show that the intake of seaweeds, which are relatively soft and easy to chew, was also reduced in the chewing group. Therefore, it would be necessary to consider offering foods that incorporate seaweeds that are familiar and affordable to the elderly in Korea.

In this study, nutrient intake tended to be similar to the food group intake, and the intake of most nutrients was significantly lower in the elderly with chewing difficulty than in the normal group. In the chewing-difficulty group, the intake of several micronutrients and macronutrients such as carbohydrates and fats was low. It is thought that insufficient intake of animal-based foods such as meat and eggs and fruits resulted in decreased vitamin and mineral intake. This result is consistent with previous studies: in the KNHANES 2015, the elderly with difficulty in mastication had lower energy and intake of protein, carbohydrate, potassium, thiamin, and vitamin C [21]. In KNHANES 2013, all nutrients except protein and vitamin A [8], and the KNHANES 2007–2010 data, showed that the intake percentage of all nutrients except vitamin A was lower than the Korean dietary reference intake (KDRI) [7]. Comparing this study with an analysis of KNHANES 2013–2018 shows nutrition insufficiency among the elderly with chewing difficulties has not improved significantly over the past 10 years. This study appears to show that the intake of fat, calcium, phosphorus, sodium, potassium, vitamin A, riboflavin, niacin, and vitamin C was significantly lower in the chewing difficulty group than in the normal group, even after adjusting for energy intake. These results indicate that chewing difficulty may decrease the quality of the diet.

Aging increases the possibility of malnutrition as food intake and the efficiencies of digestion and absorption decrease. This study also showed that the quality of meals can further deteriorate in the elderly with chewing difficulties. Although there are differences in some food groups and nutrient intake between the two groups, the nutrient intake insufficiency of the elderly has been further aggravated by chewing difficulty. In particular, the intake of calcium, vitamin A, and vitamin C was found to be far below the dietary reference intake. When comparing average nutrient intake with KDRI (recommended nutrient intake; RNI, male, 2020), calcium intake was 57.4% and 64.8%, vitamin A intake was 70.3% and 79.9%, and vitamin C intake was 67.8% and 81.8% in the chewing difficulty and normal groups, respectively. This result shows that chewing difficulty exacerbated the deficiency of nutrients important to the health of the elderly such as skeletal health, connective tissue formation, and antioxidant activity. The low intake of plant foods, such as fruits, was related to the insufficient intake of vitamin C, and the low intake of milk and dairy products was related to the insufficient intake of calcium in the chewing difficulty group. Cho [32] reported that the prevalence of osteoporosis in the elderly with fewer than 20 remaining teeth was 1.9 times higher than that of the elderly with ≥20 teeth. This result supports the efforts to encourage the consumption of calcium need to be made in the elderly with chewing difficulty.

The Japanese elderly with chewing difficulty also had a significantly lower intake of most nutrients such as protein, fat, vitamins, and minerals except carbohydrates than the elderly with no chewing problem [9], and Israeli elderly with chewing problems also reported lower energy, protein, and fiber intakes [31]. However, in the United States, except for some fat-soluble vitamins, the intake of most nutrients among the elderly with chewing difficulty was not less than normal [33]. These results show that chewing difficulty in the elderly does not necessarily lead to a poor diet, and it is possible to overcome and improve the dietary problems caused by chewing difficulty. For example, the Korean elderly are often reluctant to consume milk and dairy products due to lactose intolerance, thus it is necessary that encourage them to consume calcium with lactose-free milk and fermented dairy products such as yogurt and cheese.

In this study, poor diet and insufficient food intake caused by chewing difficulty resulted in imbalanced nutrient intake, which is thought to affect the nutritional status of the elderly.

It was found that the undernutrition of the elderly with chewing difficulty was about 1.5 times higher. Moreover, in chewing difficulty, the odds ratio of undernutrition was 1.33–1.63, which suggests that chewing ability is an important factor in the nutritional status of the elderly. Similar to these results, previous studies also reported that the chewing ability of the elderly affects nutritional status. The nutritional status score of subjects with good chewing ability was high [12,34] and the nutritional insufficiency risk was high in subjects with difficulties in chewing [7]. It has also been reported that the poor-chewing-ability group had a more than twofold rate of malnutrition compared to the good-chewing-ability group, and the chewing difficulty increased the risk of malnutrition by 1.5 times [9]. In this study, the chewing difficulty group was more likely to be underweight than the normal group, it seems that the result of undernutrition due to chewing difficulty also had an effect on bodyweight. Lee et al. [17] reported supportive results showing the negative association between chewing ability and BMI. In addition, two other studies showed an association between chewing problems and nutritional status: tooth loss led to poorer nutritional status, leading to sarcopenia [35], and the elderly with severe tooth loss had poor nutritional status with low skinfold thickness [36].

By negatively affecting the nutritional status, chewing difficulty can also affect the health-related quality of life among the elderly. As chewing ability is known to be associated with general health and wellbeing [37], it has been shown that chewing difficulty can affect mental health such as by increasing stress and depression [5,14,15,27], and lowering the quality of life [5,14]. Moreover, chewing difficulty is related to the increase in the occurrence of chronic conditions such as cardiovascular disease, musculoskeletal disease, respiratory disease, and cancer [16]. The effect on health and quality of life related to chewing difficulty in the elderly seems to be through mediating nutritional imbalance caused by poor diet. The oral health [19] and chewing ability [17,18] of the elderly reportedly affect their nutritional status and thus quality of life. In addition, the mental score of the elderly with chewing difficulty was lower, which was associated with low nutritional intake and high malnutrition [9]. The chewing ability of the elderly, through mediating nutritional status, is an important factor affecting cognitive function [12]. In the elderly with reduced chewing ability, the mortality risk increased along with the decrease in dietary diversity, in particular, the mortality risk was increased in the elderly with metabolic syndrome with chewing difficulty [11].

As observed in this study, elderly people with chewing difficulty are likely to have an unbalanced diet and, as a result, have a nutritional imbalance which could contribute to a reduced quality of life, possibly leading to degenerative disease. Therefore, it is necessary to develop food and nutrition management strategies to overcome the changes in food intake caused by chewing discomfort and to improve the quality of meals.

Intervention studies have been reported on providing a modified diet for chewing difficulties. One study prepared meals by blending and mincing to obtain a similar appearance to an ordinary meal and provided this softly cooked meal to patients with chewing problems. As a result, their intake of energy and protein increased, and patient satisfaction was high [38]. Meanwhile, in the case of providing a texture-modified, nutritious, and reshaped diet to the elderly with chewing problems, energy and protein intake were increased [39]. These results show that securing good nutritional status can be achieved by providing daily meals that consider physical aspects and nutrition tailored to the needs of the elderly with chewing difficulty. Therefore, it is necessary to study the development of soft and easy-to-swallow food for the elderly with compromised chewing ability and dental health to provide nutrients that can easily be deficient in the elderly.

In Japan, where the proportion of the elderly population is high, various processed foods, such as universal design food (UDF) and the smile care diet are provided to the elderly with poor mastication and swallowing ability. The UDF was developed with a consideration of texture and nutrition and the smile care diet was designed for supplying nutrients, and those with chewing and swallowing difficulties are provided food tailored to their needs [40]. In the United States, food for the elderly is managed by the FDA as “food for special dietary uses,” and the market supplies easy-to-chew, easy-to-swallow food and nutritional supplements for the elderly [40]. In Korea, the “Senior-friendly Industry Promotion Act” was enacted, and according to the standards for physical properties and nutrition, senior-friendly foods are being developed to make it easier for the elderly to chew and to improve their nutritional imbalances [41]. Moreover, in recent years, aging-friendly home meal replacement (HMR) products tailored to specific needs are being developed as easy-to-chew foods [42]. This shows the necessity of easily prepared meals that enable overcoming the chewing problems of the elderly.

Furthermore, Suzuki et al. [43] showed that it was possible to improve the chewing ability and nutrient intake of the edentulous elderly only with simple nutritional supports, such as dietary advice. These results suggested the need for educational strategies that can provide recommendations and guidelines to improve the diets of the elderly who experience chewing difficulty.

In conclusion, chewing difficulty aggravates nutritional imbalance in the elderly by reducing the variety and quality of diet and was found to be significantly associated with undernutrition among the elderly. As a rapidly aging society, Korean society needs customized and institutional programs that consider the oral health and chewing ability of the elderly. This development will contribute to securing the health and quality of life of the elderly by improving the quality of diet and nutritional status.

This study has certain limitations. First, since it is a cross-sectional study that analyzed KNHANES data, there is a limitation in proving causal relationships. Second, the chewing ability of each subject was judged based on their subjective response, not a medical diagnosis by dental experts. Finally, since the dietary intake was surveyed with a 24-h recall method, there is a limit to reflecting the subjects’ usual intake. Despite these limitations, however, this study is meaningful in that it provides a scientific basis for the improvement of nutritional problems caused by the elderly’s chewing difficulty and the development of health improvement programs. It is considered necessary to identify the dynamic causal relationship between the chewing ability and nutritional status of the elderly and chronic diseases in further studies. Additional research should investigate the development of senior-friendly food tailored to the needs of the elderly with chewing difficulty.

## 5. Conclusions

The purpose of this study was to investigate the relationship between chewing ability and nutritional status in the elderly in Korea. This study used data from Korean elderly aged 65 years or older who participated in the Korea National Health and Nutrition Examination Survey (KNHANES) conducted from 2013 to 2018. Of the total 7835 subjects, 43.2% had difficulty chewing. Compared to the normal group, the chewing difficulty group had more stress, less exercise frequency, less snack intake, less frequency of eating out, and a higher rate of food anxiety. It was found that the chewing difficulty group consumed significantly less food than the normal group, including various food groups such as grains, grains, potatoes, fruits, meat, milk, and dairy products. Compared to the normal group, the chewing difficulties group consumed 24.5% less fresh fruit and 17.8% less plant food (fresh fruit and nonstarchy vegetables) than the normal group. In addition, intake of most nutrients (carbohydrates, fat, calcium, phosphorus, sodium, potassium, vitamin A, riboflavin, niacin, and vitamin C) was found to be significantly lower in the chewing difficulty group than in the normal group. Finally, the result of a logistic regression analysis showed that the chewing difficulty group was significantly associated with undernutrition (OR = 1.63). In conclusion, chewing ability is closely related to food and nutrient intake in the elderly, which can reduce the quantity and quality of meals and is also related to nutritional deficiencies. Therefore, it is thought that it is necessary to develop a customized nutrition program and aging-friendly food considering the chewing ability of the elderly.

## Figures and Tables

**Table 1:** General characteristics of subjects.

Variables	Normal (n = 4378)	Chewing Difficulty (n = 3457)	Total (n = 7835)	*p*-Value ^(2)^
Chewing ability				-
Very uncomfortable	-	1065 (30.0) ^(1)^	1065 (13.0)	
Uncomfortable	-	2392 (70.0)	2392 (30.3)	
Moderate	1380 (31.3)	-	1380 (17.8)	
Not uncomfortable	1343 (30.1)	-	1343 (17.1)	
Not uncomfortable at all	1.655 (38.6)	-	1.655 (21.9)	
Gender				<0.001
Male	1973 (47.1)	1428 (42.5)	3401 (45.1)	
Female	2405 (52.9)	2029 (57.5)	4.434 (54.9)	
Age (years)				<0.001
65–74	2859 (66.3)	1922 (57.3)	4781 (62.4)	
≥75	1519 (33.7)	1535 (42.7)	3054 (37.6)	
Marital status				0.563
Married	4355 (99.5)	3434 (99.4)	7789 (99.4)	
Single	23 (0.5)	23 (0.6)	46 (0.6)	
Education Level				<0.001
Less than high-school graduate	2793 (63.6)	2599 (78.3)	5392 (69.9)	
High-school diploma	849 (21.5)	449 (13.7)	1298 (18.2)	
College degree or higher	570 (14.9)	233 (8.0)	803 (11.9)	
Region				0.005
City	3237 (78.9)	2412 (75.4)	5649 (77.4)	
Rural area	1141 (21.1)	1045 (24.6)	2186 (22.6)	
Employment status				0.221
Employed	1397 (31.7)	1046 (30.2)	2443 (31.1)	
Unemployed	2817 (68.3)	2240 (69.8)	5057 (68.9)	
Household income				<0.001
Low	1853 (41.6)	1899 (51.9)	3752 (46.1)	
Middle-low	1237 (26.9)	877 (26.5)	2114 (26.7)	
Middle-high	733 (17.6)	409 (13.7)	1142 (15.9)	
High	531 (14.0)	257 (7.9)	788 (11.3)	

^(1)^ n (weighted %) ^(2)^ *p*-value by chi square.

**Table 2:** Health-related behaviors according to chewing ability.

Variables	Normal (n = 4378)	Chewing Difficulty (n = 3457)	Total (n = 7835)	*p*-Value ^(2)^
Smoking				
Current smoker	355 (8.4) ^(1)^	402 (12.3)	757 (10.1)	
Past smoker	1257 (30.1)	998 (29.1)	2255 (29.7)	<0.001
Nonsmoker	2766 (61.5)	2056 (61.5)	4822 (60.3)	
Drinking frequency				
<1 time/month	2779 (62.2)	2320 (66.1)	5099 (63.9)	
1–4 times/month	876 (20.4)	554 (16.7)	1430 (18.8)	0.002
2–3 times/week	388 (9.2)	278 (8.5)	666 (8.9)	
≥4 times/week	333 (8.3)	304 (8.7)	637 (8.5)	
Stress level				
Severe stress	120 (2.5)	179 (5.1)	299 (3.6)	
Moderate stress	463 (11.2)	70 4(20.4)	1167 (15.2)	<0.001
Mild stress	2218 (51.3)	1648 (48.5)	3866 (50.1)	
No stress	1571 (35.1)	917 (26.0)	2488 (31.1)	
Exercise				
<1 day/week	3302 (77.0)	2788 (84.1)	6090 (80.1)	
1–2 days/week	188 (4.3)	140 (4.6)	328 (4.5)	<0.001
3–4 days/week	236 (6.0)	114 (3.5)	350 (4.9)	
≥5 days/week	490 (12.6)	243 (7.8)	733 (10.5)	
Weight				
Underweight (BMI < 18.5)	101 (2.3)	135 (4.0)	236 (3.0)	
Normal (18.5 ≤ BMI < 23)	1482 (34.3)	1203 (34.4)	2685 (34.4)	0.001
Overweight (23 ≤ BMI < 25)	1162 (27.0)	857 (25.3)	2019 (26.3)	
Obesity (BMI ≥ 25)	1613 (36.4)	1240 (36.3)	2853 (36.3)	

^(1)^ n (weighted %) ^(2)^ *p*-value by chi square.

**Table 3:** Dietary behaviors according to chewing ability.

Variables	Normal (n = 4378)	Chewing Difficulty (n = 3457)	Total (n = 7835)	*p*-Value ^(2)^
Breakfast				
5–7 times/week	1866 (93.2) ^(1)^	1544 (92.7)	3410 (92.9)	
3–4 times/week	49 (2.6)	46 (2.4)	95 (2.5)	0.849
1–2 times/week	21 (1.2)	24 (1.5)	45 (1.4)	
Rarely (<1 time/week)	52 (3.0)	46 (3.4)	98 (3.2)	
Snack				
Yes	2662 (61.3)	1930 (56.2)	4.592 (59.1)	<0.001
No	1716 (38.7)	1527 (43.8)	3243 (40.9)	
Home				
Eating	4023 (92.0)	3169 (92.1)	7192 (92.1)	0.919
Not eating	355 (8.0)	288 (7.9)	643 (7.9)	
Commercial location				
Eating	3198 (74.2)	2270 (66.4)	2215 (70.9)	<0.001
Not eating	1180 (25.8)	1187 (33.5)	2299 (29.1)	
Institution location				
Eating	273 (6.3)	229 (6.9)	502 (6.5)	0.379
Not eating	4105 (93.7)	3228 (93.1)	7333 (93.5)	
Eating-out Frequency				
≥1 time/day	230 (5.7)	138 (4.6)	368 (5.2)	
5–6 times/week	264 (6.4)	181 (5.2)	445 (5.9)	
3–4 times/week	352 (8.2)	227 (6.7)	579 (7.6)	<0.001
1–2 times/week	1197 (28.1)	813 (23.5)	2010 (26.1)	
1–3 times/month	1470 (32.85)	1119 (31.9)	2589 (32.4)	
Rarely	857 (18.73)	974 (28.2)	1831 (22.8)	
Food security				
Enough food secure	2281 (53.0)	1383 (40.9)	3664 (47.73)	
Mild food insecure	1180 (42.7)	1744 (50.6)	3624 (46.09)	<0.001
Moderate/Severe food insecure	197 (4.3)	306 (8.6)	503 (6.16)	

^(1)^ n (weighted %) ^(2)^ *p*-value by chi square.

**Table 4:** Daily food-group intake according to chewing ability.

Food Group	Normal (n = 4378)	Chewing Difficulty (n = 3457)	Total (n = 7835)	Unadjusted *p*-Value ^(2)^	Adjusted *p*-Value ^(3)^
Total Food (g)	1341.6 ± 14.6 ^(1)^	1192.4 ± 14.2	1277.1 ± 11.4	0.044	0.017
Cereals and grains (g)	284.4 ± 2.6	280.0 ± 2.9	282.5 ± 2.0	0.035	0.022
Potatoes and starches (g)	40.1 ± 2.2	34.2 ± 2.0	37.6 ± 1.6	0.021	0.012
Sugars and sweets (g)	8.0 ± 0.3	7.8 ± 0.3	7.9 ± 0.2	0.050	0.058
Legumes (g)	42.9 ± 1.4	43.5 ± 1.9	43.1 ± 1.2	0.053	0.069
Seeds and nuts (g)	8.1 ± 0.5	7.2 ± 0.6	7.7 ± 0.4	0.114	0.185
Vegetables ^(4)^ (g)	332.9 ± 4.6	297.5 ± 4.7	317.6 ± 3.5	0.365	0.191
Mushrooms (g)	4.4 ± 0.3	3.0 ± 0.4	3.8 ± 0.3	0.473	0.233
Fruits ^(5)^ (g)	209.0 ± 5.7	157.7 ± 4.9	186.8 ± 4.2	0.011	<0.001
Seaweeds (g)	33.4 ± 2.1	26.6 ± 2.3	30.4 ± 1.7	0.036	0.052
Meat and poultry (g)	58.2 ± 1.9	55.5 ± 2.7	57.0 ± 1.7	0.093	0.042
Eggs (g)	17.6 ± 0.6	13.9 ± 0.6	16.0 ± 0.5	0.020	0.057
Fishes and shellfishes (g)	98.6 ± 3.4	84.7 ± 3.7	92.6 ± 2.7	0.064	0.082
Milks and dairy products (g)	60.3 ± 2.2	52.9 ± 2.4	57.1 ± 1.7	0.023	0.029
Oils and fats (g)	4.8 ± 0.1	4.3 ± 0.1	4.6 ± 0.1	0.098	0.099
Beverages (g)	109.2 ± 4.3	96.4 ± 4.2	103.7 ± 3.1	0.049	0.062
Seasonings (g)	27.8 ± 0.5	25.6 ± 0.7	26.9 ± 0.4	0.077	0.087
Other food (g)	1.9 ± 0.3	1.6 ± 0.3	1.8 ± 0.2	0.022	0.083

^(1)^ Mean ± SE, ^(2)^ *p*-value by *t*-test, ^(3)^ Adjusted for gender, age, and energy intake, ^(4)^ Including salted vegetables, kimchi, and vegetable juice, ^(5)^ Including fruits preserved in sugar, jam, and fruit juice.

**Table 5:** Plant food intake according to chewing ability.

Plant Food	Normal (n = 4378)	Chewing Difficulty (n = 3457)	Total (n = 7835)	Unadjusted *p*-Value ^(2)^	Adjusted *p*-Value ^(3),(4)^
Fresh fruits ^(5)^	204.4 ± 5.7 ^(1)^	154.4 ± 4.9	182.8 ± 4.2	0.002	0.015
Nonstarchy vegetables ^(6)^	202.7 ± 3.8	180.4 ± 3.8	193.1 ± 2.9	0.152	0.059
Fresh fruits + Nonstarchy vegetables	407.1 ± 7.2	334.8 ± 6.6	375.9 ± 5.4	0.003	<0.001

^(1)^ Mean ± SE, ^(2)^ *p*-value by *t*-test, ^(3)^ *p*-value by the generalized linear model (GLM), ^(4)^ Adjusted for gender, age, and energy intake, ^(5)^ Excluding fruits preserved in sugar, jam, and fruit juice, ^(6)^ Excluding salted vegetables and vegetable juice.

**Table 6:** Nutrient intake according to chewing ability.

Nutrients	Normal (n = 4378)	Chewing Difficulty (n = 3457)	Total (n = 7835)	Unadjusted *p*-Value ^(2)^	Adjusted *p*-Value ^(3)^
Energy (kcal)	1724.2 ± 12.9 ^(1)^	1616.4 ± 13.8	1677.6 ± 10.4	0.216	0.092
Carbohydrate (g)	301.7 ± 2.3	287.3 ± 2.5	95.5 ± 1.8	0.042	0.053
Protein (g)	57.6 ± 0.5	52.3 ± 0.6	55.3 ± 0.4	0.519	0.521
Fat (g)	28.0 ± 0.4	24.5 ± 0.4	26.5 ± 0.3	0.057	0.035
Calcium (mg)	453.4 ± 6.1	402.0 ± 5.9	431.2 ± 4.6	0.062	0.034
Phosphorus (mg)	944.7 ± 8.7	846.2 ± 8.9	902.1 ± 6.9	0.050	0.019
Iron (mg)	14.0 ± 0.2	13.1 ± 0.3	13.6 ± 0.2	0.071	0.127
Sodium (mg)	3103.4 ± 38.6	2914.6 ± 42.2	3021.8 ± 30.2	0.027	0.047
Potassium (mg)	2792.9 ± 30.0	2479.0 ± 29.6	2657.3 ± 23.7	0.069	0.018
Vitamin A (μg RAE)	340.3 ± 9.5	293.9 ± 7.6	320.3 ± 8.7	0.034	0.045
Thiamine (mg)	1.46 ± 0.02	1.37 ± 0.02	1.42 ± 0.01	0.077	0.055
Riboflavin (mg)	1.17 ± 0.01	1.03 ± 0.01	1.11 ± 0.01	0.088	0.049
Niacin (mg)	12.1 ± 0.1	10.9 ± 0.1	11.6 ± 0.1	0.056	0.012
Vitamin C (mg)	81.8 ± 2.1	67.8 ± 1.9	75.7 ± 1.6	0.015	0.022
Energy distribution (%)					
Carbohydrate	72.7 ± 0.2	74.1 ± 0.2	73.3 ± 0.1	0.085	0.059
Protein	13.3 ± 0.1	12.9 ± 0.1	13.1 ± 0.1	0.081	0.073
Fat	14.1 ± 0.2	13.0 ± 0.2	13.6 ± 0.1	0.057	0.079

^(1)^ Mean ± SE, ^(2)^ *p*-value by *t*-test, ^(3)^ Adjusted for gender, age, and energy intake.

**Table 7:** Relationship between chewing ability and undernutrition.

	Total ^(1)^	Energy	Calcium	Iron	Vitamin A	Riboflavin
Model 1	1.630	1.421	1.361	1.570	1.272	1.398
	(1.354–1.961) ^(2),^***	(1.268–1.592) ***	(1.192–1.553) ***	(1.334–1.847) ***	(1.107–1.461) ***	(1.254–1.558) ***
Model 2	1.535	1.340	1.243	1.493	1.206	1.308
	(1.272–1.852) ***	(1.193–1.505) ***	(1.085–1.422) **	(1.266–1.760) ***	(1.050–1.387) **	(1.171–1.460) ***
Model 3	1.411	1.307	1.158	1.371	1.170	1.216
	(1.159–1.718) ***	(1.162–1.470) ***	(1.008–1.330) *	(1.155–1.628) ***	(1.015–1.349) *	(1.086–1.360) ***
Model 4	1.332	1.242	1.076	1.275	1.100	1.101
	(1.082–1.615) **	(1.101–1.401) ***	(0.919–1.259)	(1.071–1.518) **	(0.950–1.273)	(0.975–1.243)

^(1)^ Dependent variables were calculated based on the proportion of subjects consuming less than 75% of the estimated energy requirement (EER) for energy and consuming less than the estimated adequate requirement (EAR) for vitamin A, riboflavin, calcium, and iron (0: Good nutrition, 1: Undernutrition). ^(2)^ Odd ratio (95% Confidence interval) Independent variable: Chewing ability (0: Normal, 1: Chewing difficulty). Model 1: Unadjusted. Model 2: Adjusted for gender and age. Model 3: Adjusted for gender, age, smoking, drinking, stress, and weight status. Model 4: Adjusted for gender, age, smoking, drinking, stress and weight status, intake of milks and dairy products, food security, snack, eating-out frequency, breakfast, education level, household income, and marital status. * *p* < 0.05, ** *p* < 0.01, *** *p* < 0.001.

## Data Availability

All data were obtained from the Korea Disease Control and Prevention Agency and are available with the permission of the Korea Disease Control and Prevention Agency. The data in this study were from the Korea National Health and Nutrition Examination Survey.
